# Predictive Role of Biomarkers in COVID-19 Mortality

**DOI:** 10.7759/cureus.34173

**Published:** 2023-01-24

**Authors:** Ayşe Yılmaz, Öztürk Taşkın, Ufuk Demir, Veysel G Soylu

**Affiliations:** 1 Anesthesiology and Reanimation, Kastamonu University Faculty of Medicine, Kastamonu, TUR; 2 Intensive Care Unit, Kastamonu University Faculty of Medicine, Kastamonu, TUR

**Keywords:** covid-19, intensive care, systemic inflammatory response index, derived neutrophil-lymphocyte ratio, neutrophil-lymphocyte ratio

## Abstract

Background

The coronavirus disease 2019 (COVID-19) pandemic has resulted in high mortality among patients in critical intensive care units. Hence, identifying mortality markers in the follow-up and treatment of these patients is essential. This study aimed to evaluate the relationships between mortality rates in patients with COVID-19 and the neutrophil/lymphocyte ratio (NLR), derived NLR (dNLR), platelet/lymphocyte ratio (PLR), monocyte/lymphocyte ratio (MLR), systemic inflammation response index (SII), and systemic inflammatory response index (SIRI).

Methodology

In this study, we assessed 466 critically ill patients diagnosed with COVID-19 in the adult intensive care unit of Kastamonu Training and Research Hospital. Age, gender, and comorbidities were recorded at the time of admission along with NLR, dNLR, MLR, PLR, SII, and SIRI values from hemogram data. Acute Physiology and Chronic Health Evaluation II (APACHE II) scores and mortality rates over 28 days were recorded. Patients were divided into survival (n = 128) and non-survival (n = 338) groups according to 28-day mortality.

Results

A statistically significant difference was found between leukocyte, neutrophil, dNLR, APACHE II, and SIRI parameters between the surviving and non-surviving groups. A logistic regression analysis of independent variables of 28-day mortality identified significant associations between dNLR (p = 0.002) and APACHE II score (p < 0.001) and 28-day mortality.

Conclusions

Inflammatory biomarkers and APACHE II score appear to be good predictive values for mortality in COVID-19 infection. The dNLR value was more effective than other biomarkers in estimating mortality due to COVID-19. In our study, the cut-off value for dNLR was 3.64.

## Introduction

The severe acute respiratory syndrome coronavirus 2 (SARS-CoV-2) is responsible for one of the longest pandemics in world history [1]. Studies continue to reveal correlations between coronavirus disease 2019 (COVID-19) infection and diseases such as hypertension, diabetes, cardiovascular diseases, and cancer [2]. Furthermore, it has been reported that the virus affects cellular responses and has direct effects on mortality rates and causes [3].

Neutrophils are abundant in circulation, and during infection, they phagocytize microorganisms such as bacteria and fungi using neutrophil extracellular traps. However, the role and function of this mechanism during viral infections remain unclear. In postmortem examinations of patients who died from COVID-19, intense neutrophil activity was observed in the alveolar space and pulmonary capillary endothelium [4]. Increased neutrophil levels and decreased lymphocyte levels have also been observed. In particular, the ratio of absolute neutrophils to lymphocytes (NLR), calculated as neutrophil count/lymphocyte count, is significantly increased and associated with a higher risk of mortality [5,6]. A study of 245 patients with COVID-19 showed an 8% higher risk of in-hospital death for each unit increase in NLR [7].

The derived neutrophil/lymphocyte ratio (dNLR) is calculated as the neutrophil count/(white blood cell count - neutrophil count). Unlike the NLR, the difference between white blood cell and neutrophil values used in the denominator refers to monocytes and other granulocytes. Thus, NLR may better reflect the severity of infections that cause rapid increases in neutrophil production and release of poorly differentiated neutrophils [8]. The monocyte-to-lymphocyte ratio (MLR) is calculated as monocyte count/lymphocyte count, and the platelet-to-lymphocyte ratio (PLR) is calculated as platelet count/lymphocyte count.

The systemic inflammation response index (SII) was first described by Hu et al. [9], who showed that high SII scores in patients with hepatocellular carcinoma were associated with higher recurrence rates. It is calculated as neutrophil count × (platelet count/lymphocyte count). The systemic inflammatory response index (SIRI) is calculated as neutrophil count × (monocyte count/lymphocyte count) and can reflect immune and inflammatory balance [10].

In recent studies, the NLR, dNLR, PLR, MLR, SII, and SIRI have all been shown to be reliable predictors of the severity of COVID-19 infection [11,12]. Our study aimed to further evaluate the relationship between these values and mortality in COVID-19.

## Materials and methods

This retrospective study, which complied with the Declaration of Helsinki, Patient Rights Regulation, and ethical rules, was approved by the Kastamonu University Medical Research Ethics Committee (decision number: 2022-KAEK-140). Between January 2020 and January 2021, 466 critically ill patients diagnosed with COVID-19 and admitted to the adult intensive care unit of Kastamonu Training and Research Hospital were included in the study. Patient data (e.g., age, gender, and comorbidities) were collected from the hospital information management system and patient records. NLR, dNLR, MLR, PLR, SII, and SIRI values were derived from hemogram data at the time of admission to the intensive care unit. Acute Physiology and Chronic Health Evaluation II (APACHE II) values and mortality rates over 28 days were also recorded. Patients were then divided into survival (n = 128) and non-survival (n = 338) groups according to 28-day mortality. The previously described formulas were used to calculate the values [13].

Statistical calculations were conducted using SPSS version 26 (IBM Corp., Armonk, NY, USA). Continuous variables are expressed as mean ± standard deviation and compared using independent-sample t-tests. Categorical variables are described as numbers and percentages and compared using Fisher’s exact test. Finally, the predictive performance of the death indices was evaluated by estimating the area under the curve and using the corresponding receiver operating characteristic curve method.

## Results

Of the 466 patients with COVID-19 in the study, 338 (73.2%) died within 28 days of admission to the intensive care unit and 128 (26.8%) survived longer than 28 days. Analysis between groups revealed statistically significant differences in leukocyte count (p = 0.013), neutrophil count (p = 0.003), and dNLR (p = 0.003) upon admission to the intensive care unit. The non-survival group also had significantly higher APACHE II scores (23.90 ± 5.50; p < 0.001) and higher SIRI values (8.90 ± 14.79; p = 0.047). Table 1 summarizes the results.

**Table 1:** Demographic data of patients, comorbidities, hemogram parameters for admission to the intensive care unit, as well as Acute Physiology and Chronic Health Evaluation II scores, between January 2020 and January 2021.

Variable		Total (n = 466 SD)	Non-survival group (n = 338; 72.5%) Mean ± SD	Survival group (n = 128; 27.5%) Mean ± SD	P-value
Age (years)		72.03 ± 12.76	72.69 ± 12.66	70.29 ± 12.89	0.070
Gender	Female	201 (37.8%)	138 (38.3%)	63 (36.6%)	0.103
	Male	265 (62.2%)	200 (61.7%)	65 (63.4%)	
Comorbidity	Yes	352 (73.3%)	251 (73.2%)	101 (73.8%)	0.298
	No	114 (26.7%)	87 (26.8%)	27 (26.2%)	
≥2 comorbidities		173 (35.2%)	117 (34.5%)	56 (37.2%)	0.068
Diabetes mellitus		109 (22.6%)	79 (23.0%)	30 (21.5%)	0.988
Hypertension		188 (39.8%)	130 (38.9%)	58 (57.9%)	0.178
Renal disease		63 (11.5%)	45 (12.1%)	18 (9.8%)	0.953
Cardiovascular disease		131 (30.4%)	92 (30.9%)	39 (29.3%)	0.486
Respiratory disease		78 (14.2)	59 (14.5%)	19 (13.4%)	0.593
Leukocyte (10^3^/µL)		10.56 ± 4.43	10.84 ± 4.69	9.83 ± 3.56	0.013
Platelet (10^3^/µL)		214.38 ± 86.43	214.82 ± 88.77	213.23 ± 80.27	0.853
Neutrophil (10^3^/µL)		8.78 ± 4.32	9.15 ± 4.58	7.82 ± 3.35	0.003
Lymphocyte (10^3^/µL)		0.88 ± 0.63	0.87 ± 0.66	0.90 ± 0.54	0.581
Monocyte (10^3^/µL)		0.71 ± 2.82	0.73 ± 3.31	0.65 ± 0.42	0.658
Neutrophil/lymphocyte ratio		15.58 ± 17.22	16.32 ± 17.22	13.63 ± 17.14	0.132
Platelet/lymphocyte ratio		356.29 ± 347.51	365.56 ± 370.07	331.79 ± 279.16	0.290
Monocyte/lymphocyte ratio		0.87 ± 0.98	0.89 ± 1.09	0.83 ± 0.59	0.440
Derived neutrophil/lymphocyte ratio		7.10 ± 6.77	6.15 ± 0.33	8.04 ± 0.71	0.005
Acute Physiology and Chronic Health Evaluation II Score		22.54 ± 5.90	23.90 ± 5.50	18.92 ± 5.40	<0.001
Systemic inflammation response index		3,409.31 ± 4,325.28	3,582.91 ± 4,423.03	2,950.89 ± 4,036.61	0.143
Systemic inflammatory response index		8.35 ± 13.12	8.90 ± 14.79	6.90 ± 6.81	0.047

**Figure 1:**
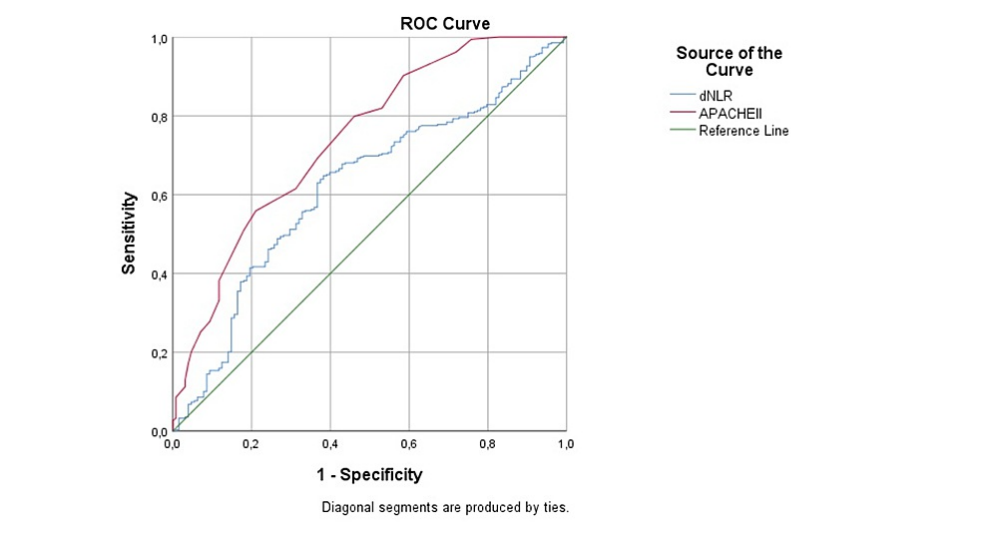
Receiver operating characteristic (ROC) curve analysis. dNLR: derived neutrophil/lymphocyte ratio; APACHE II: Acute Physiology and Chronic Health Evaluation II

A logistic regression analysis identified dNLR (p = 0.002) and APACHE II score (p < 0.001) as significant predictors of 28-day mortality. However, according to the receiver operating characteristic curve analysis, the dNLR value (area under the curve = 0.621) was found to have a low sensitivity of 70.1% and a specificity of 51.6%, with a cut-off of 3.64. Table 2 summarizes the results of the logistic regression, and Figure 1 illustrates the results of the receiver operating characteristic curve analysis.

**Table 2:** Logistic regression analysis

Variable	Β	SE	P-value	Exp(β)	95% confidence interval for Exp(β)	
					Lower	Upper
Constant	-3.573	0.547	0.000	0.028		
Derived neutrophil/lymphocyte ratio	0.066	0.022	0.002	1.069	1.024	1.115
Systemic inflammatory response index	0.009	0.010	0.371	1.009	0.990	1.028
Acute Physiology and Chronic Health Evaluation II score	0.189	0.025	<0.001	1.208	1.151	1.268

## Discussion

The results of our study showed a statistically significant difference between leukocyte, neutrophil, dNLR, APACHE II, and SIRI parameters between the survival and non-survival groups. According to the logistic regression analysis, dNLR (p = 0.002) and APACHE II score (p = <0.001) were significantly associated with 28-day mortality among participants.

Zhu et al. showed a higher mortality rate among patients with a higher white blood cell count at the time of admission in COVİD-19 patients, even when the index values were within the normal range [14]. Other studies indicate that the white blood cell value is average or decreased in COVID-19 [15,16]. In our study, a statistically significant difference was found between the survival and non-survival groups, but the white blood cell count was not found to have a substantial predictive value regarding morality.

Ghobadi et al. examined the role of NLR, PLR, MLP, dNLR, NLPR, AISI, SIRI, and SII values in predicting mortality in elderly and non-elderly patients with COVID-19. PLR, MLR, dNLR, NLPR, AISI, SIRI, and SII values were high in non-survivors (both in the elderly and non-elderly groups). The study concluded that white blood cell and neutrophil levels could be reliable predictors of mortality in COVID-19 infection [17]. The results of our study, which showed that the neutrophil value differed significantly between the survival and non-survival groups, support their conclusion. Citu et al. assessed 108 patients with COVID-19 and found that NLR, dNLR, and MLR values showed significant predictive value for mortality, but PLR and SII did not [18]. We similarly found that PLR and SII values were not significant predictors of mortality.

In a retrospective study of 807 people in Mexico with COVID-19 and acute respiratory distress syndrome, Gutiérrez-Pérez et al. found that the neutrophil to hemoglobin and lymphocyte ratio, red blood cell distribution width, as well as NLR, SII, and SIRI values, could predict severe COVID-19, the need for invasive mechanical ventilation support, and a low survival rate during hospitalization [19]. Another study by Halmaciu et al. assessed disease progression and the predictive value of the Inflammation Index (AISI) and total system score (TSS) for invasive mechanical ventilation and mortality in patients with high levels of serum interleukin 6 (IL-6) and COVID-19; they found that high MLR, NLR, SII, SIRI, AISI, IL-6, and TSS values were strong predictors of invasive mechanical ventilation and mortality [20]. Arbănaşi et al. examined predictors of thromboembolic events in COVID-19 patients and found that high MLR, NLR, PLR, SII, SIRI, AISI, and CT Severity Score values at admission accurately predicted acute lung injury, intensive care admission, and mortality [21]. Eissa et al. compared 88 patients with COVID-19 infection to 41 healthy control subjects and demonstrated that NLR >2.5, PLR >118, NLPR >0.0105, SIRI >0.8, CRP/L >7.6, and LMR <6 were essential values in the diagnosis and prognosis of COVID-19 [22]. Our study similarly found that the dNLR value was associated with mortality.

Our study had some limitations. Our research was conducted in a single center, and the study period included peak transmission and infection rates during the COVID-19 pandemic. As such, it included participants with unknown immunization status (vaccinated, partially vaccinated, and unvaccinated against COVID-19).

## Conclusions

The recent COVID-19 pandemic underscores the importance of identifying mortality markers in the follow-up and treatment of critical diseases, especially in preparation for future outbreaks. In this retrospective study of patients with COVID-19 who were admitted to the intensive care unit, inflammatory biomarkers and the APACHE II score were good predictors of mortality risk. Among NLR, dNLR, PLR, MLR, SII, and SIRI biomarkers calculated on admission, dNLR was most effective in estimating mortality related to COVID-19 disease (cut-off = 3.64; area under the curve = 0.621; sensitivity = 70.1%; and specificity = 51.6%). dNLR value is valuable as a mortality precursor in COVID-19 due to its quick and easy calculation feature.

## References

[1] Malik YS, Sircar S, Bhat S (2020). Emerging novel coronavirus (2019-nCoV)-current scenario, evolutionary perspective based on genome analysis and recent developments. Vet Q.

[2] Yan Y, Yang Y, Wang F (2020). Clinical characteristics and outcomes of patients with severe covid-19 with diabetes. BMJ Open Diabetes Res Care.

[3] Chen X, Yan L, Fei Y, Zhang C (2020). Laboratory abnormalities and risk factors associated with in-hospital death in patients with severe COVID-19. J Clin Lab Anal.

[4] Tomar B, Anders HJ, Desai J, Mulay SR (2020). Neutrophils and neutrophil extracellular traps drive necroinflammation in COVID-19. Cells.

[5] Tan L, Wang Q, Zhang D (2020). Lymphopenia predicts disease severity of COVID-19: a descriptive and predictive study. Signal Transduct Target Ther.

[6] Kouhpayeh H (2022). Clinical features predicting COVID-19 mortality risk. Eur J Transl Myol.

[7] Liu Y, Du X, Chen J (2020). Neutrophil-to-lymphocyte ratio as an independent risk factor for mortality in hospitalized patients with COVID-19. J Infect.

[8] Proctor MJ, McMillan DC, Morrison DS, Fletcher CD, Horgan PG, Clarke SJ (2012). A derived neutrophil to lymphocyte ratio predicts survival in patients with cancer. Br J Cancer.

[9] Hu B, Yang XR, Xu Y (2014). Systemic immune-inflammation index predicts prognosis of patients after curative resection for hepatocellular carcinoma. Clin Cancer Res.

[10] Usul E, Şan İ, Bekgöz B, Şahin A (2020). Role of hematological parameters in COVID-19 patients in the emergency room. Biomark Med.

[11] Peng J, Qi D, Yuan G, Deng X, Mei Y, Feng L, Wang D (2020). Diagnostic value of peripheral hematologic markers for coronavirus disease 2019 (COVID-19): a multicenter, cross-sectional study. J Clin Lab Anal.

[12] Yang AP, Liu JP, Tao WQ, Li HM (2020). The diagnostic and predictive role of NLR, d-NLR and PLR in COVID-19 patients. Int Immunopharmacol.

[13] Fois AG, Paliogiannis P, Scano V (2020). The systemic inflammation index on admission predicts in-hospital mortality in COVID-19 patients. Molecules.

[14] Zhu B, Feng X, Jiang C (2021). Correlation between white blood cell count at admission and mortality in COVID-19 patients: a retrospective study. BMC Infect Dis.

[15] Liu K, Fang YY, Deng Y (2020). Clinical characteristics of novel coronavirus cases in tertiary hospitals in Hubei Province. Chin Med J (Engl).

[16] Zhang MQ, Wang XH, Chen YL (2020). [Clinical features of 2019 novel coronavirus pneumonia in the early stage from a fever clinic in Beijing]. Zhonghua Jie He He Hu Xi Za Zhi.

[17] Ghobadi H, Mohammadshahi J, Javaheri N, Fouladi N, Mirzazadeh Y, Aslani MR (2022). Role of leukocytes and systemic inflammation indexes (NLR, PLR, MLP, dNLR, NLPR, AISI, SIR-I, and SII) on admission predicts in-hospital mortality in non-elderly and elderly COVID-19 patients. Front Med (Lausanne).

[18] Citu C, Gorun F, Motoc A (2022). The predictive role of NLR, d-NLR, MLR, and SIRI in COVID-19 mortality. Diagnostics (Basel).

[19] Gutiérrez-Pérez IA, Buendía-Roldán I, Pérez-Rubio G (2022). Outcome predictors in COVID-19: an analysis of emergent systemic inflammation indices in Mexican population. Front Med (Lausanne).

[20] Halmaciu I, Arbănași EM, Kaller R (2022). Chest CT severity score and systemic inflammatory biomarkers as predictors of the need for invasive mechanical ventilation and of COVID-19 patients' mortality. Diagnostics (Basel).

[21] Arbănași EM, Halmaciu I, Kaller R (2022). Systemic inflammatory biomarkers and chest CT findings as predictors of acute limb ischemia risk, intensive care unit admission, and mortality in COVID-19 patients. Diagnostics (Basel).

[22] Eissa M, Shaarawy S, Abdellateif MS (2021). The role of different inflammatory indices in the diagnosis of COVID-19. Int J Gen Med.

